# Cultural, Societal, and Behavioral Contributors to Delays in Seeking Care for Postmenopausal Bleeding Among Disaggregated Populations of Black Women

**DOI:** 10.3390/ijerph23050652

**Published:** 2026-05-14

**Authors:** Maurice J. Chery, Wilmar B. Mondestin, LaShae D. Rolle, Alejandra Casas, Sara M. St. George, Frank J. Penedo, Kallia O. Wright, Patricia I. Moreno, Nadine Philogene-Vincent, Sophia H. L. George, Matthew P. Schlumbrecht

**Affiliations:** 1 Department of Public Health Sciences, Miller School of Medicine, University of Miami, Miami, FL 33136, USA; mxc2759@miami.edu (M.J.C.); lashaerolle@miami.edu (L.D.R.); axc623@med.miami.edu (A.C.); s.stgeorge@med.miami.edu (S.M.S.G.); patricia.moreno@miami.edu (P.I.M.); 2 Division of Gynecologic Oncology, Sylvester Comprehensive Cancer Center, Miami, FL 33136, USA; nmp67@med.miami.edu (N.P.-V.); sophia.george@med.miami.edu (S.H.L.G.); 3 Department of Obstetrics, Gynecology, and Reproductive Science, University of Miami Miller School of Medicine, Miami, FL 33136, USA; wilmarbelizaire@gmail.com; 4 Department of Psychology, University of Miami, Miami, FL 33146, USA; fpenedo@miami.edu; 5 School of Communications, University of Miami, Miami, FL 33146, USA; kallia.wright@miami.edu

**Keywords:** endometrial cancer, postmenopausal bleeding, black women, Safer–Andersen model of total patient delay

## Abstract

**Highlights:**

**Public health relevance—How does this work relate to a public health issue?**

- Black women experience disproportionate endometrial cancer mortality, and timely evaluation of postmenopausal bleeding may be important for reducing delays in diagnosis.
- This study examines how postmenopausal bleeding is appraised and how care-seeking is anticipated across US-born Black, Caribbean-born Black, and Haitian Creole-speaking women.

**Public health significance—Why is this work of significance to public health?**

- Cultural beliefs, informal information-seeking, and structural barriers shape whether postmenopausal bleeding is recognized as serious and acted upon.
- Disaggregating Black populations reveals important nativity- and language-related differences that are often masked in aggregated analyses.

**Public health implications—What are the key implications or messages for practitioners, policy makers and/or researchers in public health?**

- Education on postmenopausal bleeding should be tailored by language, culture, and nativity to improve timely symptom recognition and care-seeking.
- Community-based navigation and communication interventions may support timely evaluation of postmenopausal bleeding, but future quantitative studies are needed to test whether these approaches reduce pre-diagnostic intervals and improve endometrial cancer outcomes.

**Abstract:**

Background: Endometrial cancer outcomes differ among Black women when examined by nativity, and timely evaluation of postmenopausal bleeding (PMB), the most common presenting symptom, may contribute to these disparities. Methods: This qualitative study explored cultural, societal, and behavioral factors shaping PMB appraisal and anticipated care-seeking among US-born Black, Caribbean-born Black, and Haitian Creole-speaking women in South Florida, guided by the Safer–Andersen Model of Total Patient Delay. Ten focus groups were conducted with 55 Black women aged ≥50 years recruited through purposive and snowball sampling. Discussions were held in English or Haitian Creole, audio-recorded, professionally transcribed, translated when needed, and analyzed thematically using a hybrid deductive–inductive approach. Reporting followed the Consolidated Criteria for Reporting Qualitative Research. Results: Three themes emerged: limited awareness and information-seeking regarding menopause and PMB; cultural and societal influences, including faith-based coping, traditional remedies, and limited family discussion of health history; and healthcare system barriers, including cost, lack of insurance, distrust, and communication challenges with providers. Subgroup differences were noted in preferred information sources, perceived susceptibility, and the role of religion in care-seeking. Conclusions: Findings suggest that PMB appraisal and anticipated care-seeking vary by nativity and language among Black women. Nativity- and language-tailored community education and navigation strategies may improve symptom recognition and support timely evaluation, but future quantitative studies are needed to test whether these approaches reduce pre-diagnostic intervals for endometrial cancer.

## 1. Introduction

Endometrial cancer (EC) is the most common gynecologic malignancy in developed countries, with rising incidence and mortality [1,2,3]. Although several EC screening strategies are under investigation [4], no approach has yet been validated for routine population-level screening among asymptomatic, average-risk women. Therefore, symptom awareness remains central to timely detection, and postmenopausal bleeding (PMB) is the cardinal symptom that should prompt clinical evaluation [5].

Black women experience disproportionately poor EC outcomes, including higher likelihood of aggressive EC subtypes, later-stage diagnosis, and higher mortality [6]. However, Black women are not a homogeneous population, and epidemiologic analyses that disaggregate Black women by nativity demonstrate substantial heterogeneity in EC burden. Non-Hispanic Black women in the United States (US) have higher incidence and poorer survival than White women overall, while subsets of Black women defined by nativity, including US-born Black, Caribbean-born Black, and Black Hispanic women, experience distinct patterns of incidence and outcomes [7]. Further disaggregation of Caribbean-born populations reveals additional heterogeneity: although overall EC incidence may be lower in some Caribbean-born groups, Haitian and Jamaican women have shown higher disease risk than US-born Black women in some analyses [8]. Among women with aggressive type 2 EC, survival is poorer for both US-Born and Caribbean-Born women compared with White women, with evidence of intra-racial heterogeneity by nativity [9]. Additional work suggests that country of birth is associated with overall survival among Black women with EC, with Haitian nativity linked to worse survival compared with some other Black subgroups, including Jamaicans [8]. These differences support the importance of examining Black subpopulations separately to better understand potential drivers of EC disparities and to inform more precise awareness and navigation strategies. These disparities unfold within a fragmented US healthcare system in which there is no national EC screening program, and diagnostic evaluation depends on patient-initiated symptom presentation followed by clinical workup [5]. Insurance coverage, specialist availability, and access to language-concordant care vary substantially and may differentially constrain Black women, particularly recent immigrants and speakers of languages other than English [10,11,12].

Timely evaluation of PMB depends not only on access to care but also on how women recognize, interpret, and act on symptoms. The Safer and Andersen model of Total Patient Delay [13] provides a useful framework for understanding this process by distinguishing several stages along the pathway from symptom recognition to treatment initiation. Appraisal delay refers to the time between noticing a bodily change and interpreting it as a symptom requiring attention. Illness delay reflects the period between recognizing a symptom as potentially concerning and deciding to seek medical care. Behavioral delay involves the time between deciding to seek care and acting on that decision, while scheduling delay captures barriers to obtaining an appointment or entering the healthcare system. Treatment delay refers to delays after diagnosis and treatment planning. These stages are often presented sequentially, but in practice they may be recursive and shaped by social, cultural, linguistic, and structural contexts.

The Safer–Andersen model has previously been applied to understand pre-diagnostic experiences among Black women with EC. In a community-engaged qualitative study of 15 Black women with a personal history of EC [14], Doll et al. used this framework to describe women’s symptom appraisal, disclosure of vaginal bleeding, and diagnostic pathways. That study provided important evidence that knowledge gaps, silence around menopause, misinterpretation of bleeding, and provider responses may contribute to diagnostic delay. However, because those participants were already diagnosed with EC, their accounts were necessarily shaped by hindsight and by knowledge that their symptoms ultimately represented malignancy. Less is known about how community-dwelling Black women without a gynecologic cancer diagnosis interpret PMB before diagnosis, or how these interpretations differ by nativity and language.

This gap is important because symptom appraisal and care-seeking are embedded within broader social and structural determinants of health. Health literacy, language access, cultural beliefs, migration history, insurance coverage, family communication, and prior experiences with healthcare systems may all influence whether PMB is normalized, monitored, discussed with others, or brought to clinical attention. These factors may operate differently among US-born Black, Caribbean-born Black, and Haitian Creole-speaking women, yet they are often obscured when Black women are analyzed as a single aggregate group. To our knowledge, no prior qualitative study has examined how community-dwelling, undiagnosed Black women interpret PMB and decide whether to seek care, with intentional disaggregation by nativity and language.

Guided by epidemiologic evidence of nativity-related differences in EC incidence and outcomes, this study qualitatively examined how cultural, behavioral, and contextual factors related to EC awareness and PMB appraisal vary across subpopulations of Black women in South Florida. Specifically, we explored how US-born Black, Caribbean-born Black, and Haitian Creole-speaking women interpret PMB, identify trusted sources of information, discuss symptoms with family or community members, and anticipate seeking care. By characterizing subgroup-specific patterns in symptom appraisal and potential care-seeking pathways, this study aims to identify actionable targets for culturally and linguistically tailored education, communication, and navigation strategies to support timely evaluation of PMB.

## 2. Materials and Methods

We conducted a community-engaged qualitative study using focus groups with three cohorts of Black women living in South Florida: US-born Black (USB, English-speaking), Caribbean-born Black (CBB, English-speaking), and Haitian-born Haitian Creole-speaking (HC). This study is reported in accordance with the Consolidated Criteria for Reporting Qualitative Research (COREQ) [15]; the completed checklist is provided as Supplementary Materials Table S1. Eligibility criteria included age ≥50 years, self-reported postmenopausal status, current residence in the US, and fluency in English or Haitian Creole. The age cutoff was selected to recruit women likely to be postmenopausal, consistent with the typical timing of natural menopause [16,17]; however, menopausal status was based on self-report and was not clinically verified. Exclusion criteria included personal history of gynecologic cancer; employment as a medical professional (e.g., nurse) with experience in gynecologic cancer; having a first-degree relative with gynecologic cancer; or inability to provide informed consent. Participants self-identified race and nativity.

The University of Miami Institutional Review Board approved the study (ID#20210626). All procedures involving human participants were conducted in accordance with the ethical standards of the institutional research committee and with the 1964 Declaration of Helsinki and its later amendments. Verbal informed consent was obtained before screening and before any study-specific procedures. Study staff explained the study purpose, procedures, risks, and participant rights in English or Haitian Creole, as appropriate. Participants were informed that participation was voluntary and that they could withdraw at any time without penalty. Participants received a $50 prepaid gift card to compensate them for their time. Sociodemographic data were collected and stored in REDCap version 15 (Vanderbilt University, Nashville, TN, USA). Audio recordings and transcripts were stored on University of Miami Box with access limited to approved study team members listed on the IRB protocol. Transcripts were de-identified before analysis, and participant codes were used in place of names or other direct identifiers. At the beginning of each focus group, facilitators reminded participants that confidentiality could not be fully guaranteed in a group setting but asked participants to respect the privacy of others and not disclose information shared during the discussion. Because discussions could involve sensitive reproductive health experiences, participants were reminded that they could decline to answer any question, pause participation, or withdraw at any time.

Using purposive [18] and snowball sampling methods [19] in partnership with community organizations, we recruited participants through radio advertisements on stations with substantial Black listenership, social media outreach, and direct contact by the study team. To reduce priming, recruitment materials described the focus groups as addressing experiences and concerns related to bleeding during menopause and did not explicitly reference EC. Participants were assigned to groups based on self-reported nativity and preferred language. Recruitment continued within each subgroup, including the smaller CBB cohort, until the team judged that thematic saturation [20,21,22] had been reached. Saturation was operationalized as the point at which additional focus groups no longer generated new codes or substantive elaborations of existing codes related to PMB appraisal, information-seeking, cultural meaning-making, or anticipated care-seeking. Saturation was assessed through ongoing review of coded transcripts and team analytic discussions after successive focus groups within and across subgroups.

Two female facilitators (WM, NPV), backed by a multidisciplinary team experienced in qualitative methods, led sessions using a semi-structured interview guide co-developed with community partners (see Supplementary Materials Table S2). Facilitators had no prior relationship with participants and introduced themselves at each session. We conducted 10 focus groups (2 in-person, 3 hybrid [Zoom + in-person], and 5 fully via Zoom). Facilitators reviewed study objectives, emphasized the value of candid feedback for shaping a cancer prevention program, and established ground rules. Focus groups ranged from 3 to 8 participants (target 5–7), proceeding with a minimum of three participants when scheduling constraints limited attendance, particularly in hybrid and virtual formats. No non-participants were present during sessions, no groups were reconvened, and field notes were not formally maintained. Sessions lasted approximately 60 min on average. Discussion prompts explored: (1) perceptions of EC risk; (2) interpretation of postmenopausal bleeding and other symptoms; (3) symptom-alleviation strategies and treatment beliefs; (4) willingness to discuss symptoms with family/community; and (5) intentions regarding care-seeking. All sessions were audio-recorded and professionally transcribed. Haitian Creole focus group recordings were professionally transcribed and translated into English. The first author, who is bilingual in Haitian Creole and English, reviewed the translated transcripts against the original language content to assess accuracy and conceptual equivalence, particularly for idiomatic expressions and culturally specific terminology related to menopause, bleeding, faith, traditional remedies, and care-seeking. Formal back-translation was not performed.

We conducted a structured, codebook-driven thematic analysis using a hybrid deductive–inductive approach. Deductive codes were informed by the Safer–Andersen Model of Total Patient Delay, operationalized as four sequential constructs. *Appraisal delay* captured how participants interpreted postmenopausal symptoms, including community misinterpretations, home remedies, and appraisal tactics such as internet searching. *Illness delay* reflected decisional weighing (e.g., uncertainty about benign vs. malignant risk, perceived severity, beliefs in treatment benefits, and negative imagery of outcomes). *Behavioral delay* captured life-context influences on readiness to act (e.g., competing demands, coming to terms with illness, and concerns about stigma). *Scheduling delay* reflected practical and structural barriers to initiating care (e.g., financial/employment constraints, home obligations, system-level hurdles, and medical mistrust). Treatment delay was not directly assessed because participants did not have a gynecologic cancer diagnosis. Inductive, data-driven codes captured concepts not prespecified by the model. Thus, the Safer–Andersen model was used as a sensitizing and organizing framework rather than as a hypothesis-testing model, allowing the analysis to remain open to culturally specific and subgroup-specific concepts not fully captured by the model.

Three co-authors (W.M., L.R., A.C.) and the first author (M.C.) independently coded each transcript, met regularly to reconcile discrepancies, and refined codes to consensus. Intercoder agreement was assessed using Cohen’s kappa, which was 0.80, indicating strong agreement. The team then organized related codes into higher-order themes and subthemes, retaining conceptually distinct processes (e.g., mistrust vs. perceived dismissal) as separate subthemes when warranted. Final codes were entered into Dedoose 10.0 (SocioCultural Research Consultants, Los Angeles, CA, USA) for data management and to examine variation by nativity/language. Using subgroup descriptors, we compared code application across cohorts, extracted key excerpts, and refined nativity-specific interpretations through team discussions.

Researcher positionality and reflexivity were considered throughout analysis. The research team included gynecologic oncology clinicians, public health researchers, behavioral scientists, and investigators with experience in cancer disparities and community-engaged research with Black, Caribbean, and Haitian communities. The first author is bilingual in Haitian Creole and English and has experience working with Haitian communities. Regular analytic meetings were used to discuss how the team’s clinical, cultural, linguistic, and disciplinary backgrounds could shape interpretation. These discussions supported reflexivity, helped surface assumptions about cultural beliefs, faith, traditional remedies, and healthcare experiences, and informed codebook refinement.

Trustworthiness was supported through several strategies. Credibility was enhanced through community-engaged recruitment, bilingual facilitation, use of multiple coders, and regular team debriefing. Dependability was supported by a structured codebook, iterative refinement of codes, and documentation of analytic decisions. Confirmability was strengthened through independent coding, intercoder agreement assessment, consensus meetings, and review of translated Haitian Creole transcripts by a bilingual investigator. Transferability was supported by providing detailed descriptions of the study setting, sampling approach, subgroup composition, and analytic procedures.

Sociodemographic data, including age, education, household income, employment status, nativity, and preferred language, were collected through a brief self-report questionnaire administered in REDCap before the focus group sessions. Demographic characteristics were summarized in SAS 9.4. [23] using means (SD) or medians (IQR) for continuous variables and counts (%) for categorical variables. Because this was a qualitative study with modest subgroup sizes, no inferential statistical comparisons were conducted. Sociodemographic data were used only to describe the sample and contextualize subgroup interpretation.

## 3. Results

Fifty-five women participated in the study, including 22 US-born Black women, 11 Caribbean-born Black women, and 22 Haitian-born Haitian Creole-speaking women. Participant characteristics are shown in Table 1. The mean age of the overall sample was 60 years. US-born Black participants had a mean age of 61.41 years, compared with 57.73 years among Caribbean-born Black participants and 60.32 years among Haitian Creole-speaking participants. Approximately 60% of the sample had attained at least a bachelor’s degree, with the highest proportion among Caribbean-born Black participants. Educational attainment differed descriptively across subgroups, with Haitian Creole-speaking participants more commonly reporting a high school education or lower. Nearly half of participants did not report income, with non-reporting most common among Haitian Creole-speaking participants. Employment status was descriptively similar across groups.

Across focus groups, participants described a progression from noticing menopausal changes to deciding whether symptoms warranted disclosure and medical evaluation. This “pathway” was shaped by three interrelated themes (Table 2): symptom knowledge and information-seeking, cultural and social meaning-making, and structural access and trust barriers. When mapped to the Safer–Andersen model, these themes corresponded primarily to appraisal, illness, behavioral, and scheduling delay. Treatment delay was not directly assessed because participants did not have a gynecologic cancer diagnosis.

### 3.1. Theme A: Limited Awareness, Misconceptions, and Information-Seeking Behaviors Shape PMB Appraisal and Potential Care-Seeking Delays

Women often began their narratives with uncertainty, describing menopause as an expected life stage but one they felt underprepared to interpret clinically. Several women noted the absence of anticipatory guidance earlier in life, and PMB was frequently framed initially through familiar bodily references, with early interpretations sometimes reducing perceived urgency.

#### 3.1.1. Subtheme 1: Recognition of Menopausal Symptoms and Misconceptions About Postmenopausal Bleeding

USB women commonly described PMB using menstrual analogies, interpreting bleeding as a continuation or variation in a known pattern rather than a distinct postmenopausal warning sign. This often appeared alongside descriptions of bodily sensations that felt like prior cycles: “I just thought it was a late period, you know” (A1-USB-2). HC women frequently described the menopausal transition as unpredictable and difficult to define, emphasizing uncertainty about timing and symptom meaning: “I myself can’t say exactly at what age… One moment I’m hot, one moment I’m sweating” (A1-HC-6). In explaining PMB, some described attributing symptoms to external or non-medical causes, which could support extended monitoring: “I believed it was something I had eaten” (A1-HC-1). CBB women tended to emphasize individual variation in menopausal timing—“Some people start at the age of 50, 45, but I believe it all depends…” (A1-CBB-3)—but more often paired this with interpretations that linked PMB to potential underlying pathology: “I believe that what causes these things are inflammation…” (A1-CBB-6). Across groups, these early appraisals shaped whether bleeding was normalized or treated as a signal warranting prompt evaluation.

#### 3.1.2. Subtheme 2: Reliance on Health Information Sources and Self-Diagnosis

Many women described a second step of gathering information outside clinical encounters before deciding whether to seek professional care for PMB. USB women frequently conducted online searching as a routine and immediate response: “Google is my buddy. Yes, I go to Google for everything” (A2-USB-1). Some described this as a means to arrive informed and prepared, but also as a form of self-triage that could delay contact with clinicians: “Yes, I’ll do research to see if anything happened before I go to the doctor so I’ll know” (A2-USB-4). HC women also described information-seeking—“If I see that I’m bleeding after menopause, I’ll always say that I’ll do research…” (A2-HC-2). CBB women similarly described online information as a preparatory tool, followed by professional consultation: “For me, I’d definitely do the research online… then I will proceed to a professional…” (A1-CBB-9), while noting that some might attempt over-the-counter management before seeking care: “So, for me, I would just go get an over-the-counter medication…” (A1-CBB-3). These accounts suggest that information seeking functioned as an intermediary stage between symptom recognition and clinical engagement, facilitating readiness to seek care but also enabling prolonged monitoring.

Taken together, Theme A mapped primarily onto appraisal delay and, to a lesser extent, illness delay. Across groups, women described uncertainty about whether PMB was normal, abnormal, or serious. However, the form of uncertainty differed by subgroup: USB women often compared PMB to prior menstrual experiences, HC women emphasized uncertainty about the menopausal transition and sometimes attributed bleeding to non-medical causes, and CBB women more often linked bleeding to possible pathology while still using online information as an intermediary step before care-seeking.

### 3.2. Theme B: Cultural Beliefs, Family Health History Awareness, and Societal Influences Shape Decisions About Disclosure and Care-Seeking

As women moved from interpreting symptoms to deciding whether to disclose or act, narratives often turned to the social and cultural contexts in which symptoms were discussed. Across groups, spirituality, family norms, and perceptions of vulnerability shaped if PMB should be shared with others, interpreted as concerning, or addressed through prayer or home-based strategies alongside, or before, medical evaluation.

#### 3.2.1. Subtheme 1: Cultural and Religious Beliefs and Family Health History Awareness

USB women framed faith as supportive and compatible with medical care, positioning prayer as a coping resource rather than an alternative to evaluation: “I’d go to the head doctor, which is Jesus Christ, God. That’s the No. 1. And then he created doctors that we have to go to, but he works through the doctors” (B3-USB-1). HC women more often emphasized divine intervention and traditional practices, sometimes describing these as initial responses to health concerns: “When you have a problem you consult God first… The greatest physician is God” (B3-HC-5), and “We Haitians… we still have little remedies… medicinal herbs” (B3-HC-7). In this subgroup, women also highlighted that limited discussion or knowledge of family history could constrain how hereditary risk informed appraisal, even when the value of such information was recognized. Caribbean-born women more often described family experiences with illness as shaping perceived susceptibility and influencing their tendency to take symptoms seriously: “Definitely, it can run in families… Right now, I have a cousin who has it…” (B3-CBB-1). These differences point to distinct ways that cultural frames and family communication practices shape how PMB is interpreted, whether symptoms are disclosed, and when medical evaluation is considered.

#### 3.2.2. Subtheme 2: Perceptions of Health Risks and Their Influence on Care-Seeking

Women described perceived vulnerability to cancer using explanations grounded in lived experience and community narratives. USB women frequently situated risk within broader social conditions, including diet and structural constraints, and emphasized that PMB and cancer were not limited by race: “I think everybody, Black or White (can develop PMB and cancer)…” (B4-USB-1), and “You (Blacks) have to get fast foods instead of cooking healthy food” (B4-USB-2). They also described selective disclosure of health concerns, suggesting that trust and fear of judgment can shape whether symptoms are shared and acted upon: “I feel, too, that the selective sharing is key…” (B3-USB-4). HC women expressed lower perceived susceptibility, describing cancer as more common among White people -“I see a lot more White people with cancer than Black people…” (B4-HC-1)—and also described privacy norms that could limit disclosure and postpone help-seeking: “I have two relatives I would not tell them. I would just pray…” (B3-HC-6). CBB women more often described PMB as potentially indicative of serious pathology—“I know it would be not something good… maybe even a tumor” (B4-CBB-3)—and described disclosure as a mechanism to mobilize support and prompt action: “I would like to tell it out to some other person…” (B3-CBB-7). These accounts suggest that perceived risk is socially constructed and can either accelerate or slow movement toward care.

Overall, Theme B reflected illness and behavioral delay processes. Cultural beliefs, perceived susceptibility, family history awareness, and disclosure norms shaped whether women interpreted PMB as requiring action and whether they would discuss symptoms with others before seeking care. HC participants more often described prayer, privacy, and lower perceived cancer susceptibility as part of symptom appraisal, while CBB participants more often described family illness experiences as cues to seriousness. USB participants emphasized selective disclosure and the need to trust the person with whom symptoms were shared.

### 3.3. Theme C: Financial and Structural Barriers, Healthcare Distrust, and System Navigation Challenges May Constrain Timely Clinical Evaluation

Even when PMB was appreciated as concerning, women described practical constraints that shaped decisions for medical evaluation. Across groups, costs, insurance barriers, and scheduling difficulties were described as central determinants of delay, while provider communication and prior experiences influenced willingness to seek care and persist through follow-up.

#### 3.3.1. Subtheme 1: Structural and Financial Barriers

USB women described the immediate financial consequences of seeking urgent care, including up-front fees and timing barriers that encouraged waiting or monitoring: “I would have to wait if it’s on the weekend… I must have $150 up front” (C5-USB-1). HC women most consistently highlighted lack of insurance or underinsurance as a primary barrier to initiating care: “Black people don’t often check the doctor’s office… Lack of insurance…” (C5-HC-2). CBB participants also described the importance of being able to access professional evaluation once symptoms were considered concerning, although structural barriers were less prominent in their narratives than among USB and HC participants.

#### 3.3.2. Subtheme 2: Trust in Healthcare Providers and Its Role in Decision-Making

Women described trust as both an outcome of past encounters and a factor influencing future help-seeking. USB women reported mixed experiences, including feeling unheard or dismissed, which contributed to provider switching and hesitancy to return promptly: “I changed doctors because I didn’t feel like my doctor was listening…” (C5-USB-2). At the same time, some expressed reliance on clinician competence and hope for diagnostic resolution once they did present: “I’m going to hope that whatever is wrong they’ll practice… to figure out what’s wrong” (C6-USB-1). HC women commonly described strong trust and adherence once connected to care, indicating that delays may occur primarily before initial clinical contact: “Anything the doctor asks me to do, that’s what I will do” (C6-HC-1). CBB women emphasized expectations of actionable next steps and solutions, which may support continued engagement once evaluated: “I’m going with a mindset that I’m going there for a solution…” (C6-CBB-9). Overall, women’s accounts suggest that communication quality and perceived responsiveness can either reinforce timely evaluation or contribute to prolonged delay through disengagement and avoidance.

Theme C mapped most closely onto scheduling delay, with some overlap with behavioral delay. Across groups, women described cost, insurance, appointment timing, provider communication, and trust as factors that could influence whether concern about PMB translated into clinical evaluation. For USB participants, prior experiences of feeling unheard or dismissed shaped trust and willingness to return promptly. For HC participants, barriers appeared most salient before initial clinical contact, particularly around insurance and access; once connected to care, participants often described high trust in clinician recommendations. CBB participants emphasized expectations for clear solutions and actionable guidance from clinicians.

## 4. Discussion

Our qualitative analysis generated distinct yet overlapping ways that subpopulations of Black women described making sense of symptoms and anticipating care-seeking for postmenopausal bleeding, processes that may shape whether bleeding is recognized as concerning and acted upon. Across all three groups, participants described (1) gaps in menopause-related knowledge and misconceptions about bleeding after menopause, (2) use of informal information sources and “research” to interpret symptoms, and (3) structural constraints that could postpone evaluation. When considered through the Safer–Andersen model, these accounts align with all four pre-diagnostic delay stages, appraisal, illness, behavioral, and scheduling, with subgroup differences most pronounced at the appraisal and scheduling ends of the pathway. Because participants were community-dwelling women without a gynecologic cancer diagnosis, our findings should be interpreted as anticipated care-seeking pathways and potential delay mechanisms rather than observed diagnostic delays.

Across groups, participants described limited menopause-related knowledge and uncertainty about what constitutes normal versus abnormal bleeding after menopause. This is consistent with prior evidence indicating that only about 63% of adults recognize PMB as a warning sign for EC [24]. However, it has been shown that when respondents are prompted with symptom lists (e.g., Womb Cancer Awareness Measure) PMB recognition increases to 80–90% [25]. This contrast indicates a gap between “knowledge when reminded” and “knowledge applied in real time.” In the Safer–Andersen model, that gap translates primarily into appraisal and illness delays; women may appreciate that PMB is abnormal but fail to label it as a problem, and attribute bleeding to benign or transient causes and continue to monitor rather than act [25]. This finding underscores the need for patient and community education that not only states that PMB is never normal, but also supports applied health literacy by helping women recognize when bleeding requires prompt clinical evaluation and how to navigate the next step in care.

Women across all three subgroups also described online searches, informal “research,” and self-diagnosis as intermediary steps between symptom recognition and care-seeking. Prior work on cancer symptom appraisal and online health information suggests that internet-based searching can both validate concerns [26] and provide benign explanations that prolong monitoring rather than prompt evaluation [27]. Rather than viewing informal information-seeking only as a barrier, these findings suggest that it may be a reachable intervention point. Culturally appropriate, language-concordant PMB messaging delivered through trusted online, community, and media channels could redirect uncertainty toward timely evaluation.

Subgroup differences were particularly evident in how women balanced faith, traditional practices, family communication, and perceived susceptibility. HC participants more often described beginning with prayer or herbal remedies, while USB and CBB participants more often framed faith as compatible with clinical care. Similar patterns have been described among immigrant and Caribbean women, where spiritual or traditional care and biomedicine often coexist, but lay remedies may be used first [28]. Haitian community research has also documented reliance on home treatments, structural barriers, and limited family-history discussion, all of which can normalize symptoms and contribute to delayed care-seeking [29]. Comparative research across Caribbean diasporas similarly shows that the sequence of lay versus formal care varies by culture and context [30]. These findings do not suggest that faith or traditional practices should be treated as inherently problematic; rather, they point to the need for interventions that respectfully integrate trusted faith, family, and community networks while clearly communicating that PMB warrants medical evaluation.

We also observed subgroup differences in perceived seriousness of PMB, with HC women more often emphasizing resilience and lower perceived susceptibility to cancer. Prior studies among Black women have described normalization of bleeding and other gynecologic symptoms as common, both of which may delay care-seeking [14]. Haitian women with EC specifically have been shown to have a high proportion of high-risk disease, presenting at advanced stages, and with worse survival [9,31]. These observations are consistent with cross-national evidence linking lower cancer awareness to longer pre-diagnostic intervals and poorer survival [32], which may compound the appraisal-stage processes described above. However, these subgroup differences should not be interpreted as cultural deficits. They likely reflect intersecting influences of language, migration history, health literacy, access to preventive information, prior healthcare experiences, and structural vulnerability.

The sociodemographic differences observed across subgroups provide further context. HC participants more commonly reported lower educational attainment and were more likely not to report income, while CBB participants had the highest educational attainment in the sample. These differences may shape access to health information, comfort navigating healthcare systems, and reliance on informal networks. For example, limited English-language access and lower exposure to culturally relevant health information may amplify the role of family, faith communities, Haitian-serving media, and community organizations in PMB appraisal among HC women. Conversely, the higher educational attainment observed among CBB participants may partly explain their greater tendency to combine online information-seeking with professional consultation. Larger quantitative studies are needed to examine how education, income, language, nativity, and migration-related factors interact to shape PMB appraisal and care-seeking.

Reports of minimization of PMB by providers among USB participants yet conditional trust in the healthcare team among HC/CBB participants are concordant with EC-specific qualitative evidence. Black patients have described practitioner responses that downplay EC risk after reporting bleeding, a mechanism authors link to scheduling and diagnostic delay whereby further evaluation is deferred [14]. In other cancers, medical mistrust has been associated with prior discrimination, suboptimal communication, and fragmented follow-up, each of which can slow progression through the Safer–Andersen pathway by reinforcing behavioral delay and contributing to repeated scheduling delays when appointments, referrals, and tests are not completed [33,34,35,36]. Within EC care pathways, conceptual and empirical work further details how provider–patient interactions and access barriers extend pre-diagnostic intervals, situating communication problems squarely within recognized delay mechanisms [37]. Our findings add nuance by suggesting that trust, mistrust, and reliance on lay care vary across Black subpopulations. For USB women, interventions may need to address prior experiences of dismissal and strengthen provider responsiveness when PMB is reported. For HC women, language-concordant navigation and insurance support may be especially important before first clinical contact. For CBB women, clear communication about diagnostic steps and expected follow-up may support continued engagement once care is initiated.

Taken together, these findings are best understood through health literacy, structural vulnerability, and intersectionality. PMB appraisal is not only an individual knowledge issue; it is shaped by the availability of understandable, culturally meaningful, and linguistically accessible health information. Structural vulnerability situates cost, insurance, language-discordant care, and fragmented access as institutional conditions that constrain timely evaluation [38], reflecting broader patterns of structural racism documented in US health systems [39]. The experience of an HC participant who interprets PMB through prayer, encounters language-discordant care, and lacks insurance is not the additive sum of “race + immigration + low income”; it is a qualitatively distinct positionality that any single-axis framework would mischaracterize [40]. These overlapping factors suggest that interventions focused only on individual awareness will be insufficient unless paired with system-level efforts to improve access, language-concordant care, and respectful clinical communication.

Limitations include single-region sampling in South Florida, which may limit transferability to Black women in other regions or to Caribbean-origin groups not represented in this study. Recruitment through community-based outreach, radio, social media, and snowball sampling may have introduced selection bias by favoring women who were more reachable, more comfortable discussing reproductive health, or more interested in health-related research. The $50 incentive may also have influenced participation, although it was intended to compensate participants for their time. Although menopausal status was confirmed by self-report, it was not clinically verified; misclassification of perimenopausal as postmenopausal status remains possible. We did not collect marital status, number of children, parity, living arrangements, caregiving responsibilities, time since migration, migration trajectory, or validated measures of acculturation, all of which may shape symptom disclosure, social support, competing demands, and care-seeking. The smaller CBB subgroup and occasional reluctance to disclose sensitive information may have limited the range of perspectives captured in that group. Mixed focus group formats, including in-person, hybrid, and virtual sessions, may have influenced group dynamics, rapport, and disclosure. Although Haitian Creole transcripts were professionally translated and reviewed by a bilingual investigator, formal back-translation was not performed, which may have limited detection of subtle translation discrepancies. Income and education heterogeneity also likely shaped perceived barriers but reflects real-world care-seeking contexts.

Strengths include rich first-person accounts, subgroup disaggregation including HC and CBB women, a community-engaged bilingual design, and theory-driven analysis using the Safer–Andersen Total Patient Delay model. Additional strengths include the focus on community-dwelling women without gynecologic cancer, which complements prior retrospective studies among women already diagnosed with EC, and the integration of subgroup-specific comparisons that can inform culturally and linguistically tailored intervention development.

## 5. Conclusions

Interpretations of PMB differed across Black subgroups and mapped to distinct potential delay patterns in the Safer–Andersen model. Because this was a qualitative study of community-dwelling women without gynecologic cancer, findings should be interpreted as potential care-seeking pathways rather than direct evidence of diagnostic delay. Results support nativity- and language-tailored education and navigation strategies that clearly communicate that PMB warrants prompt evaluation, address structural and communication barriers, and use trusted community settings such as faith communities, Haitian-serving media, community organizations, and community health centers. Future quantitative studies should test these patterns and evaluate whether tailored interventions shorten pre-diagnostic intervals and improve stage at diagnosis. Clinicians serving diverse Black populations should be supported with language-concordant communication tools and trained to elicit and counter normalization of bleeding, particularly in subgroups where faith- and family-based appraisal predominates. Scientifically, this study contributes evidence that nativity and language are not interchangeable proxies within the Black population for the purposes of understanding PMB appraisal and care-seeking.

## Figures and Tables

**Table 1:** Sociodemographic characteristics of focus group participants by subpopulation. USB = US-born Black, English-speaking; CBB = Caribbean-born Black, English-speaking; HC = Haitian-born, Haitian Creole-speaking; SD = standard deviation. Continuous variables are presented as mean ± SD; categorical variables are presented as *n* (%).

	All (*n* = 55)	USB (*n* = 22)	CBB (*n* = 11)	HC (*n* = 22)
Age in years (mean ± SD)	60 ± 6.9	61.41 ± 5.33	57.73 ± 8.32	60.32 ± 7.83
Education Attainment				
Some Post-Graduate & Graduate	13 (23.64%)	4 (18.18%)	5 (45.45%)	4 (18.18%)
Bachelor’s Degree	20 (36.36%)	10 (45.45%)	6 (54.55%)	4 (18.18%)
High School or Lower	22 (40.00%)	8 (36.37%)	0 (0%)	14 (63.64%)
Income				
Not Reported	25 (45.45%)	8 (36.36%)	0 (0.00%)	17 (77.27%)
Less than 25 K	13 (23.64%)	8 (36.36%)	4 (36.36%)	1 (4.55%)
25–49 K	3 (5.45%)	0 (0.00%)	1 (9.10%)	2 (9.09%)
50–74 K	12 (21.82%)	5 (22.73%)	5 (45.45%)	2 (9.09%)
100–249 K	2 (3.64%)	1 (4.55%)	1 (9.10%)	0 (0.00%)
Employment Status				
Employed	24 (43.64%)	6 (27.27%)	7 (63.64%)	11 (50.00%)
Retired	11 (20.00%)	7 (31.82%)	2 (18.18%)	2 (9.09%)
Unemployed or Not Reported	20 (36.36%)	9 (40.91%)	2 (18.18%)	9 (40.91%)

**Table 2:** Key themes, subthemes, and corresponding Safer–Andersen delay stages from the focus group discussions.

Themes	Subthemes	Corresponding Safer–Andersen Delay Stage(s)
A. Limited awareness, misconceptions, and information-seeking behaviors shape PMB appraisal and potential care-seeking delays	Recognition of menopausal symptoms and misconceptions about PMB	Appraisal delay
	Reliance on health information sources and self-diagnosis	Appraisal delay; illness delay
B. Cultural beliefs, family health history awareness, and societal influences shape decisions about disclosure and care-seeking	Cultural and religious beliefs and family health history awareness	Illness delay; behavioral delay
	Perceptions of health risks and their influence on care-seeking	Illness delay; behavioral delay
C. Financial and structural barriers, healthcare distrust, and system navigation challenges may constrain timely clinical evaluation	Structural and financial barriers shaping timely evaluation	Scheduling delay
	Trust in healthcare providers and its role in decision-making	Behavioral delay; scheduling delay

## Data Availability

The data presented in this study are available on request from the corresponding author.

## Supplementary Materials

The following supporting information can be downloaded at: https://www.mdpi.com/article/10.3390/ijerph23050652/s1. Table S1: COREQ checklist; Table S2: Semi-structured interview guide.

## Abbreviations

CBB	Caribbean-born Black
EC	Endometrial cancer
HC	Haitian-born Haitian Creole-speaking
IQR	Interquartile range
IRB	Institutional Review Board
PMB	Postmenopausal bleeding
SAS	Statistical Analysis System
SD	Standard deviation
USB	US-born Black
US	United States
