# Supplementary material for: Cultural, Societal, and Behavioral Contributors to Delays in Seeking Care for Postmenopausal Bleeding Among Disaggregated Populations of Black Women
Source: Int J Environ Res Public Health. 2026 May 14;23(5):652. doi: 10.3390/ijerph23050652 (PMC13205757; doi:10.3390/ijerph23050652)
Supplement: Supplementary file 1 [file ijerph-23-00652-s001.zip › ijerph-4269268-supplementary.pdf]

Supplementary Materials Table S1. COREQ Checklist

Consolidated criteria for reporting qualitative studies (COREQ): 32-item checklist

Developed from: Tong A, Sainsbury P, Craig J. Consolidated criteria for reporting qualitative research (COREQ): a 32-item checklist for interviews and focus groups. International Journal for Quality in Health Care. 2007;19(6):349–357.

Manuscript Title: Cultural, societal, and behavioral contributors to delays in seeking care for postmenopausal bleeding among disaggregated populations of Black women

No.	Item	Response	Section
**Domain 1: Research team and reflexivity**			
Personal Characteristics			
1	Interviewer/facilitator	Two female facilitators (WM, NPV) led all focus groups.	Methods
2	Credentials	Facilitators held Master of Science (MS) degrees. The broader research team included members holding PhD, MD, MPH, and MSPH degrees.	Methods
3	Occupation	Research team comprised gynecologic oncology clinicians, public health researchers, and behavioral scientists at the University of Miami Miller School of Medicine and Sylvester Comprehensive Cancer Center.	Methods
4	Gender	Both facilitators were female.	Methods
5	Experience and training	The first author and several co-authors had substantial prior experience in qualitative interviewing and focus group facilitation, as well as community-engaged research with Black, Caribbean, and Haitian communities.	Methods
Relationship with participants			
6	Relationship established	Facilitators had no prior personal relationship with participants. Participants were recruited through community organizations, radio outreach, and social media.	Methods
7	Participant knowledge of the interviewer	At the start of each session, facilitators introduced themselves, described their professional roles, and explained that the	Methods

No.	Item	Response	Section
		research aimed to inform a community-based cancer prevention program.	
8	Interviewer characteristics	Researcher positionality and reflexivity are described in the Methods. Team members’ clinical, cultural, linguistic, and disciplinary backgrounds were discussed in regular analytic meetings to surface assumptions about cultural beliefs, faith, traditional remedies, and healthcare experiences. The first author is bilingual (Haitian Creole/English) with experience working with Haitian communities.	Methods
**Domain 2: Study design**			
Theoretical framework			
9	Methodological orientation and theory	Structured, codebook-driven thematic analysis using a hybrid deductive–inductive approach. Deductive codes were informed by the Safer–Andersen Model of Total Patient Delay (appraisal, illness, behavioral, and scheduling delay); inductive codes captured concepts not prespecified by the model.	Methods
Participant selection			
10	Sampling	Purposive and snowball sampling, in partnership with community organizations.	Methods
11	Method of approach	Participants were approached through radio advertisements on stations with substantial Black listenership, social media outreach, and direct contact by the study team.	Methods
12	Sample size	55 women (22 USB, 11 CBB, 22 HC) participating in 10 focus groups.	Methods
13	Non-participation	The number of individuals who expressed initial interest but did not enroll was not systematically recorded. No enrolled participants withdrew during sessions.	Methods
Setting			
14	Setting of data collection	10 focus groups: 2 in-person, 3 hybrid (Zoom + in-person), and 5 fully via Zoom. In-person and hybrid sessions were held in	Methods

No.	Item	Response	Section
		community-accessible meeting space; virtual sessions were held by participant choice from their preferred location.	
15	Presence of non-participants	No non-participants were present during sessions.	Methods
16	Description of sample	Sociodemographic characteristics (age, education, household income, employment, nativity, preferred language) presented in Table 1, summarized overall and by subgroup.	Results
Data collection			
17	Interview guide	Semi-structured interview guide co-developed with community partners and reviewed prior to use; provided as Supplementary Material 1.	Methods
18	Repeat interviews	No focus groups were reconvened; each cohort participated in a single session.	Methods
19	Audio/visual recording	All sessions were audio-recorded and professionally transcribed.	Methods
20	Field notes	Field notes were not formally maintained; analytic observations were captured in regular team debriefing meetings.	Methods
21	Duration	Sessions lasted approximately 60 minutes on average.	Methods
22	Data saturation	Yes — saturation operationalized as the point at which additional focus groups no longer generated new codes or substantive elaborations of existing codes related to PMB appraisal, information-seeking, cultural meaning-making, or anticipated care-seeking. Saturation was assessed through ongoing review of coded transcripts and team analytic discussions.	Methods
23	Transcripts returned	Transcripts were not returned to participants for review or correction.	Methods
**Domain 3: Analysis and findings**			
Data analysis			

No.	Item	Response	Section
24	Number of data coders	Four coders (WM, LR, AC, and the first author MC) independently coded each transcript.	Methods
25	Description of the coding tree	Hierarchical coding structure organized around the four Safer–Andersen delay constructs (deductive) and inductive codes; final theme/subtheme structure presented in Table 2 with mapping to delay stages.	Methods
26	Derivation of themes	Hybrid: deductive codes derived in advance from the Safer–Andersen Model of Total Patient Delay; inductive codes derived from the data.	Methods
27	Software	Dedoose for qualitative data management and code application; SAS 9.4 for sociodemographic descriptive statistics.	Methods
28	Participant checking	Member checking with participants on findings was not conducted; preliminary interpretations were reviewed iteratively within the bilingual research team to support credibility.	Methods
Reporting			
29	Quotations presented	Yes — representative participant quotations are presented throughout the Results section, identified by alphanumeric participant codes (e.g., A1-USB-2, B3-HC-5, C6-CBB-9) indicating subtheme prompt, subgroup, and participant number.	Methods
30	Data and findings consistent	Yes — quoted excerpts directly support each theme and subtheme; subgroup comparisons are explicitly drawn.	Methods
31	Clarity of major themes	Three major themes are clearly presented (Table 2; Results, Themes A–C) with corresponding mapping to Safer–Andersen delay stages.	Methods
32	Clarity of minor themes	Subthemes within each major theme, and subgroup-specific divergent patterns (USB, CBB, HC), are described and contrasted within each theme section, with synthesis paragraphs at the close of each theme.	Methods

<u>Supplementary Materials Table S2. Semi-structured Interview Guide</u>

Script for Focus Group Session Moderator

a. **Introduction**

   Introduce moderator(s) and state the affiliation with the University of Miami. Ask participants to introduce themselves.

   Moderator: Thank you for agreeing to take part in this focus group. We appreciate your willingness to participate. As part of Sylvester Comprehensive Cancer Center's ongoing efforts to reduce disparities in women with cancer, we are conducting this focus group to inform future initiatives in communication about cancer. We need your input and want you to share your honest and open thoughts, opinions, and beliefs so that we can gather data that will be used to help how we develop our cancer prevention program.

b. **Guidelines and rules to facilitate discussion:**

   a. We want you to do the talking. We would like everyone to participate. I may call on your if I haven't heard from you in a while.

   b. There are no right or wrong answers. Every person's experiences, beliefs, and opinions are important. Speak up whether your agree or disagree. We expect and want to hear a wide range of opinions and viewpoints, and do not expect consensus, just sharing.

   c. We emphasize that what is saw in this room will remain here. You should be comfortable to share anything if sensitive issues come up. Please don't disparage another participant's remarks and let's have just one speaker at a time.

d. The discussion will last about one hour. Please silence your mobile phones. Please give everyone the chance to express her opinion during the conversation. You can address each other if you like. We are only here to facilitate and assist in the conversation.

e. We will record this session as we want to capture everything you have to say. We do not identify anyone by name in our findings. When you respond be sure to not mentor your name. You will remain anonymous. Audio recordings will be summarized and the recordings secure by the PI, Dr. Matt Schlumbrecht. We can provide summary details and findings once the study is complete.

c. Are there any questions? Then let's get started (see page with focus group questions)

d. Debrief after covering the questions.

a. Thank you for participating in the focus group today. You have provided significant insight into this issue and on behalf of the entire study team we truly appreciate your willingness to share your opinions today.

Strategies to facilitate the discussion:

- Summarize the main idea of the feedback at the end of each question
- Reflect the main idea back to the group ("Just so I understand, what you are saying is…")
- Self-appointed experts: "Thank you. What do other people think?" or "Does anybody else have a different thought or strategy?"
- The dominator: "Let's have some other comments."
- The rambler: Stop eye contact; jump in at their inhale.
- The shy participant: Make eye contact; call on them; smile at them
- The participant who talks very quietly: Ask them to repeat their responses more loudly
- Helpful probes:
    - Can you talk about that more?
    - Help me understand what you mean.
    - Can you give me an example?
- Other strategies:
    - If the conversation gets off topic, restate the purpose of the research
    - At the end indicate that this is the "final question" as this signals a summary and may generate reflective feedback

Focus Group Questions

Part 1: Perspective of personal risk

- When do you think women go through menopause or the 'change'?
- If you were to have vaginal bleeding after menopause, what do you think could be happening with your body or causing the bleeding?
- Would you be worried about the bleeding?
- If you had bleeding, would you try anything to fix it or stop it? What would that be?
- If you had vaginal bleeding after menopause, would you tell anybody?
    - If no, why not?
    - If yes, then why?
- Would you do any research about why you were having bleeding?
    - If yes, what sources would you use (eg google, social media, etc.)?
    - If no, why not?
- If you had vaginal bleeding after menopause, would you see a health care provider?
    - If no, why not?
    - If yes, then why?
        - At what point after the bleeding started **would you see a health care provider?**
- If you had vaginal bleeding after menopause, is there anything a doctor can do about it or any treatments she/he could offer?

- If you had other symptoms that occurred with the bleeding, like pain, would that make you more concerned?
    - Would any other specific symptom, if present at the same time as the bleeding, make you more likely to see a health care provider?
- What is endometrial cancer?
- Can endometrial cancer run in families?
- Are Black women at higher risk for endometrial cancer as compared to White women?
